# Gut microbial metabolites in cancer immunomodulation

**DOI:** 10.1186/s12943-025-02521-5

**Published:** 2025-12-03

**Authors:** Hengshuo Liu, Xingyu Xiong, Weizhen Zhu, Sheng Wang, Weichao Huang, Guoqing Zhu, Hang Xu, Lu Yang

**Affiliations:** Department of Urology, Institute of Urology, West China Hospital of Sichuan University, Chengdu, Sichuan Province 610041 People’s Republic of China

**Keywords:** Gut microbiota, Microbial metabolites, Tumor microenvironment, Tumor immunity, Immunotherapy

## Abstract

Gut microbiota–derived metabolites are emerging as systemic “remote immunoregulators” that shape tumor immunity across tissues. Integrating evidence across short-chain fatty acids, tryptophan derivatives, secondary bile acids, polyamines and other metabolites, we advance a metabolite–immune pathway–cancer framework that links receptor-mediated signaling, epigenetic remodeling and metabolic reprogramming to context-dependent, bidirectional immune effects. Importantly, in addition to the g protein-coupled receptor / aryl hydrocarbon receptor pathway, the selected microbial small molecule metabolites are the true T-cell receptor ligands of unconventional T cells, directly shaping the tissue resident immune and tumor microenvironment, supplementing the receptor signaling and epigenetic programs in our framework. We synthesize how these metabolites recalibrate the tumor immune microenvironment—modulating antigen presentation, T-cell effector fitness and exhaustion, regulatory T-cell activity, and myeloid polarization—and why the same metabolite can either potentiate immune surveillance or entrench immunosuppression depending on ligand–receptor pairing, dose and tissue niche. We compare tumor-type specific patterns (e.g., colorectal, liver, lung, breast and prostate cancers) to highlight common circuits and organ-restricted idiosyncrasies. Methodologically, we outline how single-cell and spatial multi-omics, imaging mass spectrometry and functional biosensors now enable co-registration of metabolite exposure with immune-cell states in human tumors, providing an actionable basis for biomarker discovery. Given ongoing debate about signals attributed to intratumoral microbiota in low-biomass tumor tissues, we foreground quantifiable, spatially mappable and pharmacologically tractable metabolite–receptor pathways, using microbe-associated molecular patterns / translocation as comparators to judge when chemical signals should be prioritized as intervention targets. Finally, we evaluate precision intervention avenues—including fecal microbiota transplantation, rational bacterial consortia, engineered microbes and nanoparticle-enabled metabolite delivery—and propose stratification rules that pair metabolite/receptor signatures with fit-for-purpose delivery. Together, mapping tissue-specific metabolite–immune circuits and embedding them in robust biomarker frameworks may convert microbial metabolites from correlative markers into therapeutic targets and tools, improving the efficacy and durability of cancer immunotherapy.

**Supplementary Information:**

The online version contains supplementary material available at 10.1186/s12943-025-02521-5.

## Introduction

Cancer continues to represent a leading global health challenge despite major therapeutic advances. Increasing evidence highlights the immunity performs a dual role in cancer: it both surveils nascent malignant cells and, when subverted, enables tumor progression through an immunosuppressive microenvironment [1–3]. Innate and adaptive immune cells—dendritic cells (DCs), natural killer (NK) cells, cytotoxic T lymphocytes (CTL)—initially provide frontline defense by detecting and eliminating transformed cells [4, 5]. Conversely, within the tumor microenvironment (TME), the expansion of regulatory T cells (Tregs), myeloid-derived suppressor cells (MDSCs), and alternatively activated macrophages establishes profound immunosuppression, undermining antitumor immunity [6–8]. Checkpoint inhibitors targeting CTLA-4 or PD-1/PD-L1 have revolutionized oncology, yet heterogeneous responses underscore the complexity of tumor–immune interactions [9–11]. 

Amid this complexity, accumulating evidence highlights the gut microbiome as a crucial determinant of cancer initiation, progression, and therapeutic efficacy [12–15]. Gut microbes shape systemic immunity through diverse mechanisms, including modulation of antigen presentation, macrophage polarization, and T-cell trafficking [16–19]. Of particular interest is the synthesis of immunoactive metabolites: short-chain fatty acids (SCFAs), tryptophan derivatives, secondary bile acids (SBAs), polyamines, and emerging mediators such as trimethylamine N-oxide (TMAO) and inosine—which act as remote immunoregulatory messengers to reprogram immune niches across tissues [20–24]. By engaging receptors such as GPR41/43 and aryl hydrocarbon receptor (AhR) or altering epigenetic programs through histone deacetylase (HDAC) inhibition, these metabolites remodel immune niches both locally and at distant tumor sites [25, 26]. In addition, select bacterial metabolites themselves function as bona fide TCR ligands for unconventional T cells, providing a chemistry-to-immunity shortcut with direct consequences for the TME and checkpoint responsiveness [27–30]. 

Notably, these metabolites display striking context-dependent duality: butyrate can simultaneously augment cytotoxic CD8⁺ T-cell activity and promote regulatory T-cell expansion; indole derivatives can either sustain antitumor Th1/CTL responses or accelerate tumor growth depending on tissue context; SBAs and TMAO have likewise been reported to exert either immunostimulatory and immunosuppressive functions depending on exposure, receptor usage and tumor type [31–34]. The same logic applies to the antigenic (TCR-ligand) axis above, where ligand class, dose and tissue inflammation bias MAIT/invariant natural killer T (NKT) cells/γδ programs toward IFN-γ-dominant versus IL-17/Th2 states [35, 36]. 

In this review, we establish a “metabolite–immune pathway–cancer” framework to critically dissect the mechanisms by which gut microbial metabolites regulate tumor immunity. We further emphasize the heterogeneity of these effects across cancer types and discuss emerging avenues for precision interventions—including fecal microbiota transplantation (FMT) rational microbial consortia, engineered bacteria, and metabolite-based delivery platforms—that may ultimately integrate microbial metabolism into personalized cancer immunotherapy.

Beyond diffusible metabolites, two cell/structure-borne microbial signals also intersect with antitumor immunity. Firstly, stress such as chemotherapy can promote selective translocation of intestinal bacteria to secondary lymphoid organs, thereby enhancing Th17/CTL responses (Cyclophosphamide mouse model) [37]. Secondly, multiple tumor types have been reported to harbor an intratumoral microbiome, where local bacterial cells and their microbe-associated molecular patterns (MAMPs) shape the TME through TLR/nucleotide-binding oligomerization domain (NOD) and other axes, affecting treatment sensitivity and even directly metabolizing chemotherapy drugs (such as gemcitabine) [38]. However, in low-biomass tissues, large re-analyses and TCGA-wide reassessments have highlighted contamination risks and batch effects, and showed that many signals diminish after stringent decontamination, cautioning against over-interpretation [39–41]. A 2025 consensus now recommends contamination-aware workflows and transparent reporting for low-biomass microbiome studies. Against this backdrop, this review center on metabolite–receptor pathways, which offer absolute quantification, spatial co-registration with immune states, and direct pharmacologic tractability, while clarify under which scenarios chemical signals should be prioritized over live bacterial/structural signals as intervention targets by comparing with the “bacterial translocation/MAMPs axis”.

## Mechanistic insights: how core gut microbial metabolites regulate tumor immunity

The tumor immune microenvironment is increasingly recognized as a dynamic interface shaped not only by host genetics and tumor-intrinsic features but also by distal microbial signals. Among these, gut microbiota-derived metabolites function as pivotal mediators bridging the intestinal ecosystem and systemic immune responses. These bioactive molecules, including SCFAs (canonical C2–C4 species and the C1 donor formate), tryptophan catabolites, SBAs, and polyamines, reach tumors through distinct anatomical routes to exert immunomodulatory effects within tumor settings [20, 42, 43]. These anatomical pathways include:

- Local/colorectal direct exposure: SCFAs and indole-derived metabolites can traverse epithelial transporters and paracellular diffusion to directly influence gut and colorectal-cancer immune function [44, 45].
- Portal-venous axis: after being produced and absorbed in the intestine, SCFAs and small polar metabolites such as TMAO enter the portal vein and undergo hepatic first-pass metabolism, establishing a portal > liver >systemic gradient that links gut-microbiome metabolites with hepatobiliary tumour immunology and systemic immune tone [34, 46].
- Bile-acid enterohepatic loop: Secondary bile acids (SBAs), converted by gut microbes, undergo enterohepatic circulation for hepatic uptake and biliary resecretion, and via FXR/TGR5 signalling and CXCL16–CXCR6⁺ NKT cell surveillance reshape tumour immunity and affect HCC and cholangiocarcinoma risk and progression [47–49].
- Lymphatic shunt: Lipophilic compounds such as polyamines and SCFAs may access intestinal lymph via chylomicron carriers and reach the systemic circulation via the thoracic duct, partially bypassing hepatic first-pass clearance; however, direct evidence for lymphatic transport of small polar metabolites remains limited [50].
- Systemic distribution: Metabolites distribute via the arterial blood to tissues and tumors; tissue exposure is determined by plasma/RBC buffering, transporter expression, local metabolism, and renal excretion. That route underlies metabolite-supplementation strategies (for example, tributyrin, inulin–propionate ester, IV/PO formate/inosine, polyamine) [51, 52].

Rather than acting as unidirectional effectors, these metabolites often display context-dependent duality, enhancing cytotoxic responses under certain conditions while fostering immune suppression in others [14, 53, 54]. Mechanistically, microbial metabolites shape cancer immunity through three principal routes:

- Epigenetic remodeling (e.g., SCFAs as HDAC inhibitors),
- Receptor-mediated signaling (e.g., GPR41/43, AhR, FXR, TGR5) see also the antigenic arm of metabolite signaling via MR1/CD1d/BTN, and.
- Metabolic reprogramming of immune and tumor cells.

**Fig. 1:**
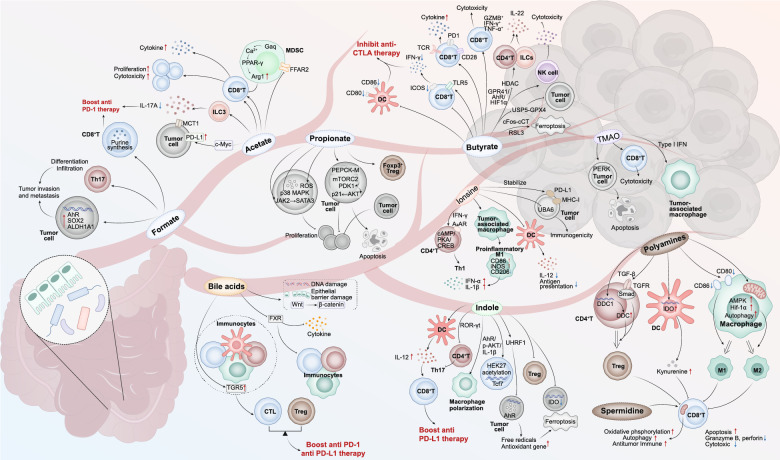
Gut Microbial Metabolites Shape Tumor Immunity via Multifaceted Mechanistic Pathways. Metabolites reach tumors through five anatomical pathways: local/colorectal direct exposure, portal-venous axis, bile-acid enterohepatic loop, lymphatic shunt, and systemic distribution, which also affect their mode of action. The key gut microbiota metabolites mediate the complex immunomodulatory networks within the TME through these anatomical routes and exert pleiotropic immune effects. Butyrate promotes CD8⁺ T-cell cytotoxicity, suppresses MDSCs and ILC3, yet enhances Tregs, with divergent impacts on CTLA-4 blockade. Acetate enhances IFN-γ⁺ CD8⁺ responses and boosts PD-1 blockade efficacy. Propionate fosters Treg and Th1 differentiation via GPR41/43 and HDAC inhibition. Formate supports folate-dependent 1 C metabolism in CD8⁺ T cells and enhances responses to anti-PD-1 therapy. Tryptophan derivatives regulate macrophage polarization and T-cell responses through AhR and STAT signaling, enhancing anti-PD-L1 activity. Spermidine modulates metabolic checkpoints (c-Myc, IDH), supports autophagy, and enhances memory T-cell development. Bile acids modulate FXR/TGR5 to control Treg and CTL balance, impacting PD-1/PD-L1 therapy outcomes. This figure integrates spatially organized pathways to depict the dualistic, tumorspecific, and therapy-influencing roles of microbial metabolites in shaping immune landscapes

These pathways converge to influence immune cell differentiation, trafficking, and effector function, ultimately conditioning the tumor immune microenvironment (Fig. 1). Importantly, the strength and direction of these effects vary across tumor types, reflecting both microbiome composition and host-specific variables.

### Microbial metabolites as direct TCR ligands for unconventional T cells

Beyond g protein-coupled receptors (GPCR) and nuclear receptors, microbial metabolites as direct TCR ligands for non-traditional T cells such as NKT cells, gamma delta T cells, and MAIT to reshape the tumor immune microenvironment in a tumor context dependent manner. Three antigen-presentation systems dominate this axis: MR1 for MAIT cells recognizing riboflavin-pathway intermediates; [27] CD1d for invariant NKT (iNKT) cells recognizing microbial glycolipids; [28] and butyrophilin (BTN3A1/BTN2A1) complexes for Vγ9Vδ2 γδ T cells sensing low-molecular-weight phosphoantigens (pAgs) [29, 30]. Collectively, these metabolite–antigen pathways influence ICI responses and reprogram myeloid compartments, nominating drug-targetable axes within the TME [55–57]. 

#### MAIT-MR1 (riboflavin-pathway ligands)

Many bacteria produce riboflavin. The reactive precursor 5-A-RU condenses with small aldehydes to generate the unstable adducts 5-OP-RU and 5-OE-RU, which bind MR1 and are presented to the semi-invariant MAIT TCR—an archetypal case of a microbial metabolite serving as the antigen [58]. The amount of ligand and the surrounding inflammatory milieu tune MAIT programs toward either IFN-γ/granzyme–dominant cytotoxicity or IL-17–skewed responses, helping to explain why MAIT cells can bolster antitumor immunity in some settings yet dampen NK/T-cell surveillance in others [59, 60]. In melanoma, MAIT deficiency improves NK-dependent control of metastasis, highlighting this context dependence, whereas in colorectal cancer (CRC) models MAIT activation can be protective by remodeling innate compartments [60]. 

#### iNKT-CD1d (microbial glycolipids)

Commensal and environmental bacteria such as *Sphingomonas* synthesize α-linked glyco-/glycosphingolipids that load onto CD1d and are recognized by the iNKT TCR [61]. Subtle differences in the carbohydrate headgroup and acyl chains tune iNKT output—skewing responses toward Th1-like IFN-γor Th2/IL-4 [62]. In tumors, rapid iNKT activation can license DCs, counter suppressive myeloid programs, and provide adjuvanticity that augments PD-1/PD-L1 blockade or vaccines [56, 63]. This structure–function bias is recapitulated with next-generation, CD1d-loaded glycolipid agonists.

#### Vγ9Vδ2-BTN (phosphoantigens)

Many bacteria and parasites use the MEP/DOXP (non-mevalonate) pathway to generate low–molecular-weight pAgs such as HMBPP [64]. Inside target cells, these metabolites bind the B30.2 domain of BTN3A1 and act as a ‘molecular glue’ that stabilizes BTN3A1–BTN2A1 complexes, triggering inside-out activation of the Vγ9Vδ2 TCR [30, 65]. The result is a rapid cytotoxic and IFN-γ–rich response. Translationally, agonistic antibodies to BTN2A1 can amplify this circuit in vitro and in vivo, promote forms of immunogenic tumor cell death and inflame otherwise ‘cold’ tumors, thereby complementing PD-1/PD-L1 blockade [66]. 

Taken together, chemically specified microbial metabolites, rather than generic MAMPs, can act as antigens that license unconventional T cells. The dose, tissue distribution, and inflammatory state determine whether MAIT/iNKT/Vγ9Vδ2 responses augment cytotoxic surveillance or skew toward suppressive circuits, thereby modulating ICI sensitivity and myeloid reprogramming.

### Short-chain fatty acids

SCFAs, including acetate, propionate, butyrate, and the microbiota-derived one-carbon donor formate represent the most extensively studied class of microbial metabolites in cancer immunity. Beyond their classical roles as energy substrates for colonocytes, these molecules remodel epigenetics, receptor signaling and cellular metabolism. Canonical C2–C4 SCFAs can energize CD8⁺ T cells and condition DCs, yet may also induce tolerance via HDAC inhibition and myeloid free fatty acid receptor 2 (FFAR2) signaling, underscoring context-dependent effects [67, 68]. Canonical SCFA actions also are chain-length and cell-type specific: butyrate/pentanoate enhance activated CD8⁺ T-cell programs, whereas propionate does not increase CD8⁺ memory potential and can curb priming indirectly by dampening DC IL-12/IL-23 [69]. Distinct from C2–C4 SCFAs, formate acts primarily as a folate-dependent one-carbon (1 C) donor to sustain de novo purine synthesis in tumor-infiltrating CD8⁺ T cells and thereby augment PD-1 blockade in vivo. In some cases, formate can also support oncogenic programs. This highlights the need for SCFAs to play a role in dose, tissue, and source aware translation.

#### Formate

Microbiota-derived formate has recently been identified as a functionally consequential gut–immune metabolite that connects microbial one-carbon (1 C) output to antitumor T-cell metabolism. In murine tumors, exercise reshapes the microbiota toward higher formate production, elevates luminal/systemic formate, promotes Tc1 polarization of CD8⁺ T cells, and potentiates anti–PD-1 efficacy; gain- and loss-of-function experiments support that formate is both sufficient and required for these benefits [70]. Mechanistically, stable-isotope tracing demonstrates that exogenous formate is assimilated by tumor-infiltrating CD8⁺ T cells into the folate-dependent 1 C network, supplying 10-formyl-THF for de novo purine synthesis and thereby sustaining clonal expansion and effector programming under checkpoint pressure [71]. Beyond physiologic sources, pharmacologic 1 C supplementation—including intravenous formate and oral methanol as a formate pro-drug (with deuterated variants for kinetic control)—raises circulating formate and synergizes with anti–PD-1, markedly increasing the durability of tumor regressions in MC38 and other models [52]. Converging evidence now indicates that supplying one-carbon units with formate restores metabolic fitness of tumor-infiltrating CD8⁺ T cells and augments the antitumor activity of PD-1 blockade [72]. 

Context matters for formate biology. Tumor cells with active mitochondrial one-carbon (1 C) metabolism can release excess formate, in some settings, fuels disease progression [73, 74]. In CRC, Fusobacterium nucleatum supplies formate that activates AhR signaling and stemness programs, thereby enhancing invasion. These data caution that strategies leveraging formate must account for tumor- and microbiota-derived sources and their divergent effects [75]. Translationally, these data nominate formate as a tractable SCFA-class metabolite that does not primarily act via FFAR2/3 or HDAC but instead fuels nucleotide biosynthesis in T cells [52]. Two complementary routes merit evaluation—behavioral prehabilitation (exercise) to enrich high-formate microbial output, and timed 1 C-donor regimens (formate salts or methanol pro-drugs) co-administered with ICI—while prioritizing dose/schedule optimization, T-cell-directed delivery, and biomarker-guided selection (plasma/fecal formate; microbial 1 C-pathway signatures) to mitigate tumor-metabolic co-benefit. These observations align with the review-wide theme that route, tissue accumulation, and source specificity often dictate net immunologic directionality.

#### Acetate

Acetate illustrates the dual nature of SCFAs in tumor immunity. Foundational studies show that acetate programs T-cell responses by supplying acetyl-CoA to metabolic–epigenetic nodes: during systemic stress or infection, acetate boosts memory CD8⁺ T-cell recall via ATP-citrate-lyase–dependent GAPDH acetylation, increasing glycolytic reserve and IFN-γ output [76]. Under glucose restriction resembling the TME, acetate uptake and acetyl-CoA synthetase–dependent metabolism restore histone acetylation and chromatin accessibility at effector loci (e.g., Ifng), rescuing cytokine production; more broadly, the coupling of acetyl-CoA availability to histone acetylation underpins Th1 differentiation and IFN-γ transcription [77, 78]. Consistent with these mechanisms, acetate can support CD8⁺ T-cell function by serving as a substrate for histone acetylation and mitochondrial oxidation, thereby promoting proliferation and effector activity [78–80]. *Bacteroides thetaiotaomicron*-derived acetate promoted M1 macrophage polarization, enhanced CD8⁺ cytotoxicity, and restrained tumor growth in hepatocellular carcinoma (HCC) models, with epigenetic up-regulation of ACC1 in macrophages [81]. On the other hand, tumor cells can capture acetate to generate acetyl-CoA via ACSS2, upregulating c-Myc and driving proliferation, accompanied by PD-L1 upregulation and immune evasion [82, 83]. Moreover, acetate accumulation within the TME can activate FFAR2 signaling in MDSCs, inducing an Arg1 dependent, L-arginine depleting program to impair cytotoxic T-cell function; pharmacologic inhibition of FFAR2 alleviated MDSC-mediated immunosuppression and enhanced immune-checkpoint blockade in preclinical models [53]. These divergent effects highlight ongoing controversies: systemic acetate levels and their impact on cancer progression remain inconsistent across tumor types, and even strain-specific microbial sources can elicit opposite outcomes. Such complexity suggests that acetate could serve as a biomarker of immunotherapy response, but therapeutic modulation requires caution: systemic supplementation could enhance effector T-cell responses but simultaneously fuel tumor metabolism. Targeting transporters (e.g., MCT1) or metabolic enzymes (e.g., ACSS2) might improve therapeutic selectivity.

#### Propionate

Propionate exerts both antitumor and immunosuppressive functions. It can inhibit tumor cell proliferation and induce apoptosis in colorectal and breast cancer models by inducing cell cycle arrest and activating stress-related pathways, while also enhancing CD8⁺ T-cell cytotoxicity [14, 84, 85]. Building on T-cell–intrinsic mechanisms, seminal work shows chain-length specificity: butyrate and pentanoate enhance CTL/chimeric antigen receptor (CAR)-T function via metabolic/epigenetic reprogramming, whereas acetate or propionate do not increase the memory potential of activated CD8⁺ T cells, underscoring context dependence [69]. Beyond direct T-cell effects, propionate modulates antigen-presenting cells (APC): in human monocyte-derived DCs, propionate (together with butyrate) reduces IL-12/IL-23 secretion and thereby diminishes MART-1–specific CD8⁺ T-cell activation; exogenous IL-12 rescues this effect, and acetate has no such activity [86]. 

Conversely, propionate fosters differentiation of peripheral Foxp3⁺ Tregs by HDAC inhibition [87]. It also acts on lymphocyte lineages other than alpha beta T cells, directly inhibiting the production of IL-17/IL-22 by gamma delta T cells through HDAC dependent mechanisms, and jointly maintaining the immunosuppressive network in the TME [88]. Clinical evidence further associates high circulating propionate with resistance to anti–CTLA-4 therapy, suggesting that its immune-modulating role may compromise checkpoint efficacy [89]. These findings underscore the dose -dependent and model-specific effects of propionate on tumor and immunity, which may be considered a double-edged metabolite: it could be leveraged as an adjuvant to chemotherapy or radiotherapy by inhibiting tumor proliferation, yet whose manipulation in immunotherapy requires careful patient stratification and monitoring of circulating levels.

#### Butyrate

Butyrate has attracted particular attention for its pleiotropic effects on tumor immunity. It enhances CD8⁺ T-cell cytotoxicity and synergizes with anti–PD-1 therapy in colorectal and lung cancer models, partly through HDAC inhibition, regulation of T-cell exhaustion pathways, and modulating T cell receptor signaling [90–93]. Butyrate can not only improve and modulate the function and differentiation of NK cells, B cells, macrophages and DCs through a variety of ways [94–96], but also augment chemotherapy efficacy and induce ferroptosis, thereby promoting long-term antitumor memory [14, 97–102]. Yet paradoxically, butyrate may also drive immune tolerance. In murine anti-CTLA-4 models, butyrate impairs DC maturation and T cell ICOS expression, limiting expansion of tumor-specific and memory T cells and attenuating antitumor efficacy [89]. It downregulates mTORC1 and c-Myc in NK cells, reduces activating receptors and cytokine secretion, diminishing cytotoxicity [103], and exacerbates T cell exhaustion in CAR-T therapy via HDAC inhibition [104]. These divergent effects illustrate that butyrate’s impact is highly context-dependent, shaped by tumor type, systemic vs. local metabolite concentration, and host microbiota composition. Such complexity creates both obstacles and opportunities. Butyrate holds promise as a therapeutic adjuvant, particularly in combination with immunotherapy and chemotherapy. However, patient stratification based on butyrate-producing microbiota or serum butyrate levels will be crucial. Moreover, strategies to control butyrate dosage, timing, and delivery mode (e.g., targeted delivery vs. systemic supplementation) will be required to avoid paradoxical immunosuppression.

#### Pentanoate

Although less extensively studied than acetate, propionate, and butyrate, valerate and branched-chain SCFAs are emerging as modulators of tumor immunity. Preclinical studies indicate that pentanoate enhances the antitumor activity of CTLs and CAR T cells through coordinated metabolic and epigenetic reprogramming [69]. Metabolic tracing further suggests that pentanoate improves T-cell adaptability and reduces exhaustion; unlike butyrate or propionate, it does not promote regulatory T-cell differentiation in the CD4⁺ compartment [105]. In a gut-on-chip model recreating the human intestinal adenocarcinoma microenvironment, pentanoate, more so than acetate, preferentially drives a proinflammatory Th17 phenotype and fails to diminish markers of T cell exhaustion [104]. Current evidence remains fragmentary, and discrepancies likely stem from differences in microbial producers, host diet, and tissue-specific accumulation patterns. These uncertainties underscore the need for systematic evaluation of valerate in well-controlled clinical settings. If validated, valerate could emerge as a complementary metabolite for modulating T-cell immunity or as part of engineered microbial consortia designed to boost immune responsiveness while minimizing tolerance.

### Tryptophan associated microbial metabolites

Tryptophan catabolism in the gut proceeds mainly via the indole pathway, yielding indole, indole-3-lactic acid (ILA), indole-3-pyruvic acid (IPyA), indole-3-propionic acid (IPA), and indole-3-acetic acid (3-IAA) [106]. These metabolites act as potent immunomodulators by engaging the AhR, remodeling chromatin states, and regulating oxidative metabolism [107, 108]. Recent work demonstrates that microbial indoles can also activate AhR in tumor-associated macrophages (TAMs), driving immune suppression and accelerating pancreatic cancer growth [109]. 

ILA has garnered extensive attention for its anticancer properties. Beyond its reported anti-proliferative activity in CRC cells [110], ILA enhances IL-12 secretion by DCs through chromatin remodeling, thereby priming CD8⁺ T-cell immunity against tumor growth; ILA also improves the function of tumor-infiltrating CD8⁺ T cells [111]. Additional studies reveal that ILA modulates Th17 differentiation and macrophage polarization through the AhR/p-AKT/IL-1β axis [112, 113]. At the systems level, ILA from *L. gallinarum* can cooperate with the indoleamine 2,3-dioxygenase (IDO)1/kynurenine/AHR axis to improve anti-PD-1 efficacy by limiting Treg activity in CRC, providing a mechanistic link between ILA-centered epigenetic tuning of DCs and checkpoint responses [111]. 

In contrast, IPyA, an intermediate in tryptophan metabolism, exhibits context-dependent, dualistic effects. In breast cancer, *Prevotella copri* reduces host IPyA levels and thereby promotes tumor growth via UHRF1 mediated phosphorylation of AMP-activated protein kinase (AMPK) [114]. Conversely, tumor-intrinsic IPyA can act as an AhR ligand to promote HCC progression and glioblastoma migration [115, 116]. Mechanistically, IPyA and other indole derivatives also act as free radical scavengers, upregulating antioxidant programs to suppress ferroptosis and support tumor survival [117]. 

As an immunotherapy adjuvant, IPA sustains stem-like properties of CD8⁺ T cells by promoting H3K27 acetylation at the Tcf7 super-enhancer, thereby enhancing responsiveness to anti-PD-1 therapy across multiple cancer models [107]. ICA, a partial AhR agonist, inhibits IDO1to reduce kynurenine production and Treg infiltration, thereby enhancing anti-PD-1 efficacy [45]. Clinically, the microbial metabolite 3-IAA has been found enriched in pancreatic ductal adenocarcinoma (PDAC) patients who respond to chemotherapy; FMT, dietary tryptophan modulation, or oral 3-IAA supplementation enhanced chemotherapy efficacy in humanized mouse models [118]. However, further clinical studies indicate that the predictive role of 3-IAA may vary across cohorts and disease stages [119]. 

Collectively, tryptophan-derived metabolites exert pleiotropic and often contradictory effects: they can potentiate CD8⁺ T-cell responses and improve immunotherapy efficacy, yet also fuel tumor progression via myeloid-AhR activation or ferroptosis suppression. This duality highlights their potential as both biomarkers of treatment response and targets for selective AhR modulation.

### Secondary bile acids

Gut microbiota remodel primary bile acids into a diverse pool of SBAs that signal through the nuclear receptor FXR and the GPCR TGR5 to regulate tumor immunity and microenvironment. Beyond the liver’s classic glycine/taurine conjugation, microbial bile salt hydrolases not only remove host conjugates but also add amine or amino-acid groups, generating many microbially conjugated bile acids (MCBAs) [120–122]. Conjugation increases polarity and favors transporter-dependent uptake, which tilts signaling toward membrane receptors such as TGR5 [123]. In contrast, deconjugated, oxo- and iso-epimeric species more readily enter cells to act on nuclear receptors including FXR, RORγt and vitamin D receptor [124–126]. Large-scale mass-spectrometry surveys show that MCBA diversity is far greater than previously appreciated, and several MCBAs can directly activate TGR5 and FXR, positioning bile acid conjugation as a tunable microbial node in anti-tumor immunity [48, 121]. At the organismal level, a microbiome–bile acid–immune axis can control liver tumor growth by tuning hepatic chemokines and C-X-C chemokine receptor (CXCR) 6⁺ NKT-cell accumulation, underscoring tissue specificity that complements receptor-driven effects observed across cancers [48]. 

Functionally, deoxycholic acid (DCA) inhibit Toll-like receptor-4-mediated activation of splenic and intestinal macrophages, suppress secretion of IL-6, IFN-γ, and TNF-α, and drive polarization of antitumorigenic M1 macrophages toward a pro-tumorigenic M2 phenotype [33, 127, 128]. In vivo, DCA directly impairs CD8⁺ T-cell cytotoxicity via the PMCA–Ca²⁺–NFAT2 axis, promoting colorectal tumor growth [33]. In HCC, taurolithocholic acid (TLCA) fosters an immunosuppressive niche by polarizing TAMs toward M2 phenotypes, which supports tumor-initiating cell maintenance [129]. Counterbalancing these effects, ursodeoxycholic acid (UDCA) triggers TGR5–cAMP–PKA–CHIP–dependent phosphorylation and autophagic degradation of TGF-β, thereby restraining Treg differentiation and synergizing with anti–PD-1 therapy to induce durable antitumor responses [130], and UDCA reshapes the gut microbiota, enriching *Akkermansia*, to protect against colitis-associated carcinogenesis [131]. 

Conjugation tends to steer secondary bile acids toward signaling through the membrane GPCR TGR5; notably, TLCA and lithocholic acid (LCA) are among the most potent endogenous TGR5 agonists [124, 132]. ໿Deconjugated and chemically modified SBAs (for example, oxo- or iso-epimers) more readily enter cells and act on nuclear receptors [125]. In T cells, derivatives of LCA act as selective immune modulators. 3-oxoLCA binds the transcription factor RORγt and restrains Th17 differentiation. By contrast, isoalloLCA promotes induction of Foxp3⁺ Tregs via mitochondrial reactive oxygen species (ROS) and Foxp3 upregulation; oral dosing reproduces these effects in vivo [124]. A related secondary bile acid, isoDCA, increases peripheral Tregs by dampening dendritic-cell stimulatory programs, a response linked to FXR signaling in DCs. Together, these pathways couple microbial bile-acid chemistry to the Th17–Treg balance, a key determinant of sensitivity to immune-checkpoint blockade. These chemistry-driven preferences help explain why immune outcomes differ across tissues and cell types. Receptor context helps explain divergent outcomes across tissues. In non-small cell lung cancer (NSCLC), FXR acts as a tumor-promoting driver, transactivating IL-6 and IL-6ST and activating the Jak2/signal transducer and activator of transcription 3 (STAT3) pathway, thereby enhancing metastatic potential and poor prognosis [133]. At the same time, TGR5 signaling in TAMs supports M2-type polarization via the cAMP–STAT3/STAT6 axis, suppressing CD8⁺ T-cell functions [134]. By contrast, hepatic models reveal FXR’s protective facets in bile-acid homeostasis and tumor suppression, consistent with organ-specific functions along the enterohepatic axis [48, 135]. 

Together, these data establish SBAs as context-dependent immunomodulators: DCA-dominated pools can compromise cytotoxic T-cell function and favor protumor myeloid programs, whereas UDCA skews toward immune restoration and immune checkpoint blockade (ICB) sensitization. Therapeutically, ligand- and receptor-selective strategies—e.g., modulating FXR/TGR5 in specific cell types, or profiling bile-acid signatures to stratify patients—may help exploit beneficial axes (e.g., UDCA or microbiome engineering) while avoiding SBAs-driven immune tolerance.

### Polyamines

Polyamines—including putrescine, spermidine, and spermine—are abundant in many tumors and are also supplied by the gut microbiota via decarboxylation of arginine/ornithine. In the intestinal lumen, polyamines are also supplied by specific commensals. Representative producers include *Enterobacteriaceae* such as *Escherichia coli* (harboring the canonical polyamine genes), *Enterococcus faecalis* (agmatine-deiminase operon *aguBDAC*), *Bacteroides* spp. (e.g., *B. thetaiotaomicron* using carboxyspermidine routes), *Bifidobacterium adolescentis*, *Lactobacillus plantarum*, and several *Clostridium/Lactobacillus* lineages [23, 136–139]. These taxa convert arginine or ornithine to agmatine and putrescine, and then to spermidine through conserved pathways. Because microbial production coexists with intrinsic synthesis (ODC1/AMD1/SRM/SMS) in epithelial, immune, stromal, and tumor cells, source attribution should integrate metagenomic detection of spe/agu modules, gnotobiotic/antibiotic controls, and/or stable-isotope tracing to separate microbial from host origin [140–142]. 

Within the TME, elevated polyamines modulate immunity in a context-dependent manner. On the suppressive side, polyamines promote regulatory T-cell differentiation and stabilize lineage programs in T cells [143, 144]. In conventional DCs, an “Arg1→polyamine→IDO1” relay establishes a durable tolerogenic state that depletes tryptophan and accumulates kynurenines to restrain CD8⁺ T-cell priming [145–147]. In macrophages, spermidine activates mitochondrial superoxide–AMPK–HIF-1α signaling and autophagy, down-modulates costimulatory molecules, and polarizes cells toward an M2-like phenotype, thereby dampening antitumor responses [148, 149]. In animal models, polyamines induces CD8 + T cell apoptosis, downregulate granzyme B and perforin to reduced cytotoxic function, and drive tumor invasiveness in an immune dependent manner [150]. 

Conversely, spermidine can bolster antitumor immunity under defined immunometabolic states. As the obligate substrate for eIF5A hypusination, it facilitates the translation of mitochondrial proteins essential for oxidative phosphorylation and supports autophagy and mitophagy in immune cells [151]. In aged mice, dietary spermidine re-activated fatty-acid oxidation of T cell and synergized with anti-PD-L1 to improve antitumor activity [152]. These bidirectional effects likely reflect dose, timing, tissue compartment, and immune context (e.g., aged versus inflamed TME) rather than a simple “good–bad” dichotomy.

Polyamine-blocking strategies are now in human testing and reported at SITC/JITC 2024, with first-in-human safety/pharmacodynamic readouts [153]. Multiple ongoing trials (e.g., NCT06465199; NCT05717153) are assessing polyamine-blocking in advanced solid tumors and CNS malignancies with metabolic/immune endpoints. Taken together, we propose a biomarker-guided framework: in polyamine-rich, Arg1/IDO1-dominant TMEs, prioritize polyamine/transport blockade and relief of arginine–tryptophan bottlenecks; in aged or energetically constrained immunity, consider physiologic spermidine support to restore eIF5A-dependent mitochondrial programs with careful dosing and immune checkpoint inhibitor (ICI) combinations.

### MAMPs and bacterial translocation

Unlike small-molecule metabolites that readily diffuse across compartments, MAMPs, such as lipopolysaccharide (LPS), peptidoglycan (PGN), muropeptides and flagellin, signal through pattern recognition receptors (PRRs; for example, TLRs and NOD-like receptors) to trigger APC-driven priming, cross-presentation and cytokine programs that can either potentiate or blunt antitumor immunity [154, 155]. The biological outcome depends on the chemical structure of the MAMP (for example, lipid A acylation state), the anatomical context of exposure (gut–lymphoid translocation versus intratumoral colonization), and the host sensor engaged (for example, NOD2 versus NOD1). This section contrasts these “cell/structure-bound” cues with diffusible metabolites to delineate scenarios in which MAMP-centric interventions may be prioritized [37, 156, 157]. 

#### Bacterial translocation to secondary lymphoid organs and tumors

Cytotoxic chemotherapy can remodel the intestinal barrier and microbiota, enabling selective translocation of Gram-positive commensals to mesenteric lymph nodes and spleen. In mouse models, cyclophosphamide provokes gut bacterial translocation that elicits pTH17 and Th1 responses and supports cytotoxic T-cell activity; ablation of the microbiota or loss of these responses diminishes therapeutic efficacy [37]. Enterococcus hirae and Barnesiella intestinihominis are among the taxa functionally linked to this effect, with E. hirae–specific memory Th1 responses associating with clinical benefit in patients receiving chemotherapy [158]. 

Checkpoint immunotherapy itself can also promote controlled translocation: anti-PD-1/anti-CTLA-4 regimens in mice induced movement of endogenous gut bacteria to tumor-draining lymph nodes and subcutaneous melanomas, where microbial sensing augmented antitumor T-cell responses [159]. Moreover, systemically delivered Bifidobacterium can accumulate intratumorally and convert non-responders into responders to CD47 blockade via STING-dependent type I IFN signaling, illustrating a mechanistic bridge between distant gut communities and local therapy responses [15]. 

#### Intratumoral microbiota as local MAMP sources

Tumors harbor low-biomass, tumor type–specific intracellular bacteria within both cancer and immune cells. Rigorous multi-omic and imaging pipelines have mapped these consortia across cancers (for example, breast, lung, melanoma), linking bacterial presence and predicted functions to clinical features and treatment response [39, 160]. Functionally, intratumoral Gammaproteobacteria can metabolize gemcitabine via a long isoform of bacterial cytidine deaminase (CDDL), conferring drug resistance that is reversible with targeted antibiotics—an archetypal example of local microbes altering therapeutic exposure and efficacy [38]. In PDAC, distinct tumor microbiomes stratify long- versus short-term survivors, in part through immune-modulatory programs [161]. Together, these studies underscore that where and how bacteria (and their MAMPs) are encountered matters.

#### Structure function rules for MAMPs

The immunologic polarity of LPS hinges on lipid A acylation [162]. Recent patient-linked and preclinical data identify hexa-acylated LPS (typical of Enterobacteriaceae) as a potent toll-like receptor 4 (TLR4) agonist that is required for full anti-PD-1 efficacy; oral hexa-acylated LPS boosts intratumoral CD8⁺ T-cell effector function and tumor control, whereas penta-acylated LPS (common in Bacteroidota) fails to enhance therapy and can antagonize hexa-LPS–induced activation [156]. These findings support structure-aware LPS profiling and caution against indiscriminate antibiotic or endotoxin-modulating interventions in immunotherapy candidates.

Gut-derived LPS engages TLR4 along the hepatobiliary axis to promote cholangiocarcinoma (CCA). In causal mouse models that mimic clinical risk states (primary sclerosing cholangitis or colitis), increased gut permeability raised portal LPS, which activated hepatocyte TLR4, induced CXCL1, and recruited CXCR2⁺ polymorphonuclear MDSCs, thereby establishing an immunosuppressive niche that accelerates CCA [49, 163]. Genetic disruption of the hepatocyte TLR4–CXCL1 arm similarly reduced MDSCs and tumor burden, positioning this pathway as a tractable driver of disease. At the tumor-cell level, LPS–TLR4 signaling drives migration/invasion via a METTL3–PI3K/AKT axis—an effect attenuated by pharmacologic or genetic interference—and translationally nominates companion measurements and a druggable node for combination with checkpoint blockade or myeloid-reprogramming [164, 165]. 

Beyond LPS, MAMP specificity at NOD sensors yields divergent outcomes: Enterococcus peptidoglycan remodeling via the secreted hydrolase SagA generates muropeptides that activate NOD2, enhance cross-presentation, and consistently improve anti-PD-L1 responses in multiple tumor models [166, 167]. By contrast, systemic NOD1 activation can expand MDSCs and sustain Arg1-dependent suppression, fostering tumor-permissive microenvironments in CRC models [168]. MAMPs also vary across tumors: in the esophagus, Bacteroides LPS can drive TLR4/MyD88/NF-κB signaling and epithelial mesenchymal transition, whereas intestinal Prevotella-derived LPS has been linked to inflammatory tolerance that restrains colorectal tumorigenesis in vivo, emphasizing that tissue context and lipid A chemistry co-determine net outcomes [169, 170]. 

Metabolite-driven pathways provide tunable, systemic levers that can be delivered or inhibited without relocating live bacteria; these often synergize with checkpoint blockade by conditioning T-cell metabolism and APC programs. By contrast, MAMP/translocation axes act as punctate, contextual “danger” cues whose benefits are maximized when (i) structural agonism is favorable (for example, hexa-acylated LPS), (ii) exposure can be localized (tumor or draining nodes), and (iii) the engaged PRR skews toward antitumor immunity (for example, NOD2 over NOD1 in CRC). Thus, in patients with intact mucosal barriers and metabolite signatures predictive of response, metabolite-first strategies may be preferable; when barrier perturbation (chemo/ICI) or intratumoral colonization is expected, MAMP-aware adjuvants or decolonization strategies merit consideration. Because low-biomass signals can arise from reagent and cross-sample contamination, large re-analyses and TCGA-wide surveys have questioned many claimed tumor-resident microbes; thus, any functional inference from intratumoral bacteria or their MAMPs must meet contamination-aware and orthogonally validated criteria (negative controls and blanks across the workflow, quantitative burden exceeding blanks, and orthogonal visualization/qPCR/in-situ chemistry) [40, 41, 171–173]. 

### Other gut microbial metabolites

Beyond SCFAs, bile acids, indoles and polyamines, several additional gut–microbiota–derived metabolites shape tumor immunity in a context-dependent manner and increasingly intersect with clinical immunotherapy.

#### Trimethylamine N-oxide

TMAO, a product of dietary choline metabolism by commensals, shows bidirectional actions. In tumor-promoting settings, it can activate oncogenic signaling (e.g., Wnt/β-catenin, ILK/AKT/mTOR, MAPK), induce inflammation/oxidative stress, and enhance proliferation, angiogenesis and metastasis [174–177]. Conversely, in the context of immunotherapy, TMAO sensitizes tumors to ICB: in triple-negative breast cancer, higher intra-tumoral/plasma TMAO associates with an activated immune microenvironment, and TMAO triggers PERK-dependent ER stress/pyroptosis to strengthen CD8⁺ cytotoxicity and improve anti-PD-1 efficacy; [178] in PDAC, TMAO upregulates type-I IFN regulators, promotes immunostimulatory TAMs and effector T cells, and synergizes with anti-PD-1/anti-Tim-3 to reduce tumor burden and extend survival [34]. Clinically relevant caution comes from cellular therapy: broad-spectrum antibiotics that deplete TMAO-producing commensals lower circulating TMAO and impair anti-CD19 CAR-T efficacy [179]. Together, TMAO illustrates a metabolite whose benefit or harm is state-matched (tumor type, immune context, concomitant therapy).

#### Inosine

Microbiota-derived inosine is a context-dependent immunomodulator. With IFN-γ co-stimulation and A₂A-receptor engagement, inosine can support Th1 differentiation and potentiate CTLA-4/PD-L1 blockade in murine tumors [24]. Beyond receptor signaling, when glucose is scarce in the TME, effector T cells can use inosine as fuel. Purine nucleoside phosphorylase converts inosine into ribose-1-phosphate and hypoxanthine: the ribose feeds glycolysis and the pentose phosphate pathway, while hypoxanthine enters the purine-salvage pathway to maintain nucleotide supply, sustain proliferation, and preserve IFN-γ production [180]. Complementing this salvage route, formate supplementation drives de novo purine synthesis in tumor-infiltrating T cells and augments anti-PD-1 efficacy in vivo, underscoring a metabolic axis that can synergize with inosine support [52]. In adoptive cellular therapy, inosine rewires CAR-T metabolism and epigenetic stemness, increasing potency and persistence [181]. Beyond T cells, inosine can push TAMs toward a proinflammatory M1 state and augment TNF-α/IL-1β, but it can also limit DC antigen presentation and IL-12 production via A₂A signaling, blunting naïve T-cell priming [182, 183]. However, A₂A engagement on effector T cells may also elevate cAMP and dampen IFN-γ production, proliferation, and cytotoxicity, potentially protecting tumors from clearance [184]. These data support inosine as a double-edged mediator: it can fuel and expand antitumor T-cell programs while A₂A-mediated signaling remains immunosuppressive, net effect depends on receptor context, costimulation, and route/dose of delivery. Rational combinations, such as metabolic inosine support paired with A₂A blockade or ex vivo conditioning, offer a path to partition benefits from adenosinergic suppression.

## Comparative landscape of microbial metabolite immune interactions in solid tumors

Across solid tumors, gut microbiota–derived metabolites operate as context-dependent immunomodulators, tuning antitumor or protumor programs through receptor engagement and epigenetic/metabolic rewiring of immune cells. Notably, the same metabolite can play divergent roles across tissue and immune context, exemplifying the intricate and sometimes paradoxical dialogue between host immunity and microbial chemistry. To capture this cross-cancer heterogeneity, we adopt a “metabolite–immune pathway–tumor” framework for the cross-cancer comparison (Table 1), emphasizing biomarker-guided stratification and precision interventions to rationally pair metabolites with immunotherapies.

**Table 1:** Gut microbiota metabolites and Immunomodulatory mechanisms across tumors

Tumor Type	Effect			Metabolite		Specific Mechanism		References	
Colorectal Cancer									
	Promoting tumor progression			DCA		Antagonizes FXR; expands Lgr5⁺ CSCs; induces DNA damage; activates TGR5/STAT3, Wnt/β-catenin, NF-κB pathways		[33, 185–187]	
				TMAO		Enhances VEGFA secretion; induces inflammation, oxidative stress, DNA damage		[176]	
				Spermidine, Putrescine		Upregulates IL-6, IL-17; drives Th17 polarization; creates immunosuppressive environment		[188]	
	Inhibiting tumor progression			SCFAs		HDAC inhibition; activates GPR41/43; promotes Foxp3⁺ Tregs; enhances CD8⁺ T-cell cytotoxicity		[14, 87, 91, 98]	
				ILA		Modulates macrophages via AhR-p-AKT-IL-1β; suppresses cytokines; induces apoptosis		[110, 112, 113]	
	Enhances immunotherapy efficacy			Formate		Fuels one-carbon/purine synthesis in CD8⁺ T cells → ↑proliferation/effector molecules; synergizes with anti-PD-1 in preclinical CRC models		[52, 70, 71]	
				Butyrate		Promotes CD8⁺ T-cell infiltration; synergizes with anti-PD-1 therapy		[91]	
				Indole-3-carboxaldehyde		Inhibits Treg differentiation; modulates IDO1-kynurenine-AhR axis; enhances CD8⁺ T cell activity		[45]	
Hepatocellular Cancer									
	Promoting tumor progression			DCA		Inducing DNA damage; releasing pro- tumorigenic factors; triggering mitochondrial ROS → EGFR/MAPK; activates CXCL16/LSEC → CXCR6 ⁺ NKT axis		[48, 189, 190]	
				LCA		Activates cAMP → STAT3/STAT6 and inflammation immune suppression pathway; and DCA coordinates the shaping of a tumor promoting microenvironment		[48, 191]	
				TMAO		Activates ILK/AKT/mTOR; impairs CD8⁺ T cell infiltration		[177]	
	Inhibiting tumor progression			Acetate		1.Activates GPR43; inhibits IL-6/JAK1/STAT3; prevents NAFLD-associated HCC 2.Promotes M1 macrophage polarization; enhances CD8⁺ T cell function via histone acetylation and ACC1		[81, 192]	
				Butyrate		Disrupts Ca²⁺ homeostasis; induces ROS and cancer cell death via JNK/p38 MAPK		[193]	
				IPA		Stimulates γδ T cells to produce granzyme B/perforin; boosts CD8⁺ T-cell-mediated therapy response		[107, 194]	
				D-lactate		Reprograms TAMs from M2 to M1 via PI3K/Akt inhibition and NF-κB activation		[195]	
Lung Cancer									
	Promoting tumor progression			Acetate		Activates FFAR2-Gαq/Ca²⁺/PPAR-γ/Arg1 in MDSCs		[53]	
				TLCA		Activates TGR5/cAMP-STAT3/STAT6; drives M2 polarization		[134, 196]	
				DCA		trans-activates CCND1 via FXR		[197, 198]	
	Inhibiting tumor progression			Butyrate		HDAC inhibition; enhances ID2; promotes CD8⁺ cytotoxicity; GPR43-Foxp3-PD-L1 axis		[14, 92, 98]	
				UDCA		Suppresses TGF-β; blocks MAPK; induces apoptosis		[199]	
Breast Cancer									
	Inhibiting tumor progression			Butyrate		1.Reverses EMT; inhibits MEK/ERK pathway 2.Inhibits Wnt/β-catenin via LRP5 downregulation; HDAC inhibition; H3K9 acetylation; impair cancer stemness		[200, 201]	
				IPA		↑H3K27ac at Tcf7 enhancer in CD8⁺ T cells; represses UHRF1; preserving antitumor immune microenvironment		[107, 114]	
	Enhances immunotherapy efficacy			Acetate		Enhances CD8⁺ T cell proliferation and function; induces antitumor immunity		[79, 80]	
				TMAO		Induces pyroptosis via PERK; enhances CD8⁺ T-cell immunity and immunotherapy response		[34]	
Prostate Cancer									
	Promoting tumor progression (castration-resistant prostate cancer)			Acetate, Butyrate		TLR3-NF-κB/MAPK-CCL20 → M2 macrophage infiltration; ↑IGF1 → MAPK/PI3K		[202, 203]	
	Promoting tumor progression			TMAO		Activates p38 MAPK pathway; upregulate HMOX1; proliferation, migration		[174]	
	Inhibiting tumor progression			Urolithin A		Enhances NK cell activity via AhR pathway		[204]	

The effects and mechanisms listed in this table are derived from experimental studies in cellular, animal, or clinical models. Reference numbers correspond to citations in the main text

*AhR* Aryl hydrocarbon receptor, *CSCs* Cancer stem cells, *DCA* Deoxycholic acid, *EMT* Epithelial-mesenchymal transition, *FXR* Farnesoid X receptor, *HDAC* Histone deacetylase, *IDO1* Indoleamine 2,3-dioxygenase 1, *IGF1* Insulin-like growth factor 1, *IL* Interleukin, *IPA* Indole-3-propionic acid, *JNK* c-Jun N-terminal kinase, *LCA* Lithocholic acid, *MAPK* Mitogen-activated protein kinase, *MDSCs* Myeloid-derived suppressor cells, *NF-κB* Nuclear factor kappa-light-chain-enhancer of activated B cells, *NK cells* Natural killer cells, *PERK* Protein kinase RNA-like endoplasmic reticulum kinase, *PBMCs* Peripheral blood mononuclear cells, *ROS* Reactive oxygen species, *SCFAs* Short-chain fatty acids, *STAT3* Signal transducer and activator of transcription 3, *TAMs* Tumor-associated macrophages, *TLR* Toll-like receptor, *UDCA* Ursodeoxycholic acid, *TMAO* Trimethylamine N-oxide, *Tregs* regulatory T cells, *UHRF1* Ubiquitin-like with PHD and RING finger domains 1

### Colorectal cancer

Across CRC, microbial metabolites fall into context-dependent immunosuppressive “drivers” and immuno-restorative “modulators”. SBAs, most notably DCA, can antagonize intestinal FXR, expand Lgr5⁺ cancer stem–like cells, destabilizes genomes, and activate oncogenic programs including TGR5/STAT3, Wnt/β-catenin, and NF-κB cascades, thereby skewing epithelial and immune programs toward carcinogenesis [33, 185–187]. Consistent with these observations, LCA-derived metabolites rewire the Th17–Treg axis: 3-oxoLCA restrains Th17 differentiation, whereas isoalloLCA and isoDCA promote peripheral Tregs [124, 125]. These chemistry-defined effects suggest that SBAs profiles could help stratify responsiveness to immune-checkpoint blockade in CRC. In vitro, TMAO enhances VEGFA secretion and CRC cell proliferation; excessive exposure further accelerates tumorigenesis via inflammation, protein misfolding, oxidative stress, and DNA damage [176]. Likewise, polyamines such as spermidine and putrescine up-regulate pro-inflammatory cytokines (IL-6, IL-17), skew CD4⁺ T-cell differentiation toward a Th17 phenotype, and thereby establish an immunosuppressive microenvironment conducive to tumor growth, while polyamine-targeting strategies can restore antitumor immunity [188, 205].

In contrast, certain microbial metabolites exert potent antitumor immune effects on CRC. SCFAs, classically acetate/propionate/butyrate, and the newly recognized one-carbon donor formate cooperate but act through distinct immunometabolic logics. Butyrate, propionate, and acetate suppress HDAC and activate GPR41/43 receptors to promote Foxp3⁺ regulatory T-cell differentiation, inhibit NF-κB-mediated inflammation, and strengthen CD8⁺ T-cell cytotoxicity, collectively restraining CRC progression [14, 87, 91, 98]. Whereas microbiota-derived formate fuels 1 C/purine synthesis in tumor-infiltrating CD8⁺ T cells, enhancing effector fitness and checkpoint responsiveness [52, 90]. Indole derivatives (e.g., ILA) can reprogram macrophage polarization via AhR-linked signaling, dampen pro-tumor cytokines, and reduce proliferation while promoting apoptosis [110, 112, 113]. Beyond diffusible metabolites, ligand quality within microbial-structural signals also matters: hexa-acylated LPS from commensals more potently licenses anti-PD-1 responses than penta-acylated LPS in CRC models [156]. Enterococcus SagA-derived NOD2-stimulatory muropeptides augment anti-PD-L1 control in MC38 [166]. Conversely, myeloid NOD1 sustains Arg1⁺ MDSCs and drives colorectal carcinogenesis; and under cyclophosphamide, Gram-positive commensals translocate to lymphoid tissues in CT26-bearing mice to amplify Th17/CTL priming [37, 168]. 

Therapy interface: in vivo, butyrate accelerates CD8⁺ T-cell infiltration and synergizes with anti-PD-1 in CRC models, consistent with SCFAs’ HDAC-dependent T-cell programming [14, 91]. Branched SCFAs such as isobutyrate show similar potentiation in CT26 [206]. Intravenous formate or oral methanol as a formate pro-drug increase formate availability to tumor-infiltrating lymphocytes and augment anti-PD-1 efficacy in MC38, aligning with the 1 C-metabolic constraint of effector T cells [52]. 

Moreover, indole-3-carboxaldehyde enhances anti-PD-1 efficacy in CRC by inhibiting CD4⁺ Treg differentiation and modulating the IDO1-kynurenine-AhR axis to bolster CD8⁺ T-cell function, enhances anti-PD-1 efficacy in CRC [45]. FXR agonism by allocholic acid reprograms oncogenic signaling and restrains CRC growth in preclinical systems, whether it consistently sensitizes tumors to immune checkpoint blockade requires further evaluation [207]. Collectively, these findings illuminate a bidirectional regulatory axis in which gut microbial metabolites orchestrate CRC biology and treatment sensitivity via multifaceted immune mechanisms.

### Hepatocellular carcinoma

Gut microbiota-derived metabolites are transported to the liver via the portal vein, the gut–liver axis makes HCC uniquely sensitive to microbially produced small molecules that tune intrahepatic immunity. On the protective signals side, acetate from *Bifidobacterium pseudolongum* activates GPR43 and suppresses IL-6/JAK1/STAT3 signaling, and alleviates NAFLD-HCC–prone inflammatory tone, while *Bacteroides thetaiotaomicron*-derived acetate promotes M1 macrophage polarization and enhances CD8⁺ T-cell function in the liver [81, 192]. Considering the exposure of the gut liver axis and portal vein to gut derived metabolites, the potential value of formate enhanced anti-PD-1 response in HCC models is worth considering. Butyrate perturbs intracellular Ca²⁺ homeostasis, increases ROS, and triggers JNK/p38 MAPK-dependent HCC cell death, and it enhances sorafenib efficacy in vivo [193]. The tryptophan metabolite IPA stimulates γδ T cells to release high levels of cytotoxic cytokines, such as granzyme B and perforin, thereby suppressing HCC progression [107, 194]. Beyond SCFAs and indoles, d-lactate reprograms pro-tumor M2 TAMs into anti-tumor M1 TAMs by inhibiting the PI3K/Akt pathway and activating NF-κB, converting a “cold” TME into an immunologically “hot” state [195]. 

On the tumor-promoting side, SBAs (e.g., LCA, DCA) promote HCC progression [48, 134, 190]. Intestinal derived DCA induces DNA damage and age-related secretory phenotypes in hepatic stellate cells, releasing pro-inflammatory and pro tumor mediators [189]. In liver cells, DCA increases mitochondrial reactive oxygen species, thereby activating oncogenic EGFR/MAPK signaling [190]. In addition, as a secondary bile acid, DCA also reduces the expression of CXCL16 in sinusoidal endothelial cells, limits the accumulation of CXCR6 in NKT cells, and weakens liver immune surveillance [48]. LCA (and its high affinity conjugate TLCA) as a strong TGR5 agonist activates cAMP → STAT3/STAT6 and the inflammatory immunosuppressive pathway in the liver that bias macrophages toward immunosuppressive states, which establishes a pro-tumor microenvironment conducive to HCC progression [48, 191]. 

In the context of bile acid recirculation through the portal vein, conjugated species tend to signal through the membrane GPCR TGR5, whereas unconjugated or chemically modified bile acids more readily enter cells and activate nuclear receptors; these chemistry-dependent routes can differentially shape intrahepatic myeloid and T-cell responses across the enterohepatic axis [48, 124]. Additionally, TMAO facilitates HCC by activating the ILK/AKT/mTOR axis and impairing CD8⁺ T-cell infiltration and effector function [177]. Not all tryptophan fluxes are beneficial: in Myc-driven liver tumors, IPyA formation is an oncometabolic requirement; dietary Trp restriction suppresses tumor growth by limiting IPyA synthesis [115]. Along the gut–liver axis, diffusible metabolites can be titrated systemically, whereas translocated MAMPs act as localized “danger” cues. TLR4 engagement by gut-derived LPS promotes HCC in chronically injured livers and can induce neutrophil extracellular traps [208, 209]. While obesity-induced lipoteichoic acid translocation activates TLR2 on hepatic stellate cells to amplify IL-1β/IL-33 SASP programs and Treg-mediated immunosuppression [210, 211]. And NOD signaling in liver TAMs shows immune-activating potential [212]. 

These metabolite–immune axes have translational leverage in HCC: acetate/SCFAs and D-lactate findings support metabolite or microbiome-based adjuvants to sensitize the liver TME; [81, 192, 195] IPA enhances CD8 ⁺ T cell-mediated anti-PD-1 therapeutic response in multiple tumor types [107, 194]. And structure matters for LPS, as hexa-acylated (but not penta-acylated) LPS enhances anti-PD-1 responses across tumor models [156]. 

### Lung cancer

Gut microbiota-derived metabolites have a significant impact on the occurrence and development of lung cancer. Clinical and mechanistic data indicate that SCFAs shape lung-tumor immunity in a cell-type–dependent manner [213]. In anti-PD-1–treated cohorts, responders with NSCLC display higher circulating acetate, propionate and butyrate; mechanistically, butyrate increases H3K27ac at PDCD1 and CD28 regulatory regions, augmenting TCR signaling and cytotoxicity, thereby potentiating anti-PD-1 therapy [92]. Ex vivo, sodium butyrate reduced viability and induced apoptosis in NSCLC patient-derived epithelial cells, while increasing peripheral CD4⁺ T cells [95]. Acetate has been shown to promote progression by activating FFAR2 and triggering the Gαq/Ca²⁺/PPAR-γ/Arg1 signaling pathway in MDSC, drives an immunosuppressive phenotype that promotes tumor growth in lung adenocarcinoma models [53]. Thus, untargeted systemic SCFAs supplementation may have opposing effects; receptor- or cell-selective strategies (e.g., FFAR2 antagonism in MDSCs versus boosting butyrate programs in CD8⁺ T cells) merit consideration. In tumor-infiltrating macrophages, LPS/TLR4 signaling sustains NF-κB -driven IL-10 production and dampens effector T-cell activity, while intratumoral bacteria are prevalent (including in lung cancers) and correlate with immunotherapy response—highlighting a pattern-bound axis that can oppose or re-shape metabolite effects [39, 214, 215]. SBAs also bifurcate outcomes in NSCLC, FXR drives metastasis by transactivating IL-6ST/IL-6 and activates JAK2/STAT3, with higher FXR tumors showing worse prognosis and preclinical sensitivity to FXR inhibition [133, 197]. Conversely, TGR5 deficiency restrains M2 macrophage polarization and activates antitumor immunity [134, 196]. Clinically actionable counter-examples exist: UDCA augments doxorubicin efficacy in NSCLC by suppressing TGF-β/MAPK and triggering apoptosis. These data argue for subtype- and receptor-guided BA interventions in lung cancer [199]. 

Beyond SCFAs and SBAs, emerging tryptophan-derived signals now link gut commensals to lung tumor control. *Bifidobacterium animalis* was recently shown to suppress NSCLC progression via its metabolite 3-IAA, which modulates AhR–m⁶A/STAT3 signaling, reshapes macrophage, and boosts CD8⁺ T-cell function along the gut–lung axis [216]. Dosing and timing remain critical, while butyrate can potentiate PD-1 blockade, systemic SCFAs may dampen CTLA-4 efficacy or NK function in specific contexts, arguing for biomarker-guided, compartment-aware modulation in lung cancer.

### Breast cancer

Microbial metabolites orchestrate breast cancer initiation, progression, and therapeutic response through diverse immune and stemness pathways. For example, acetate enhances effector T cell function and proliferation, restoring CD8⁺ T-cell-mediated antitumor immunity in murine breast cancer models [79, 80]. Beyond acetate, SCFAs with stronger HDAC-inhibitory activity exhibit anti-metastatic signals in breast cancer models. Sodium propionate suppresses proliferation and promotes apoptosis in xenografts via the JAK2/STAT3/ROS/p38-MAPK axis, both propionate and butyrate suppress breast cancer cell migration and invasion by reversing epithelial-mesenchymal transition and inhibiting the MEK/ERK pathway [85, 200]. Yet metabolite effects are compartment- and dose-dependent: acetate can fuel tumor-intrinsic c-Myc–dependent PD-L1 upregulation and immune evasion in other solid tumors, underscoring the need for cell-selective or receptor-selective delivery [82]. 

The tryptophan–indole branch in breast cancer appears to act primarily through tumor-intrinsic metabolic restraint rather than direct T-cell programming: expansion of *Prevotella copri* depletes host IPyA, relieving UHRF1-mediated repression of AMPK and thereby promoting tumor growth; restoring IPyA re-engages AMPK signaling and slows progression [107, 114]. This dovetails with observations that psychological stress–induced gut dysbiosis can activate LRP5/β-catenin–dependent cancer stemness in breast tumors, reinforcing the rationale for microbiota-metabolite strategies that simultaneously support antitumor immunity and constrain stemness programs [201]. In triple-negative breast cancer (TBNC), TMAO induced pyroptosis in tumor cells by activating the endoplasmic reticulum stress kinase PERK, enhanced CD8 T cell-mediated antitumor immunity in vivo, and correlated with improved efficacy of immunotherapy [178]. 

Breast cancer harbor intracellular bacteria in both malignant and immune cells, with tumor-type–specific signatures that correlate with immunotherapy response [39]. Functionally, intratumoral bacteria promote metastatic colonization in spontaneous breast-tumor models [217]. Moreover, LPS–TLR4 signaling in breast-cancer cells can activate NF-κB and pro-invasive programs, while TAM-derived IL-10 sustains immunosuppression—motivating co-profiling of metabolites and MAMP pathways (e.g., TLR4 activity) when interpreting immune phenotypes or designing combinations [218]. 

### Prostate cancer

Microbial metabolites shape PCa growth and the tumor–immune ecology in a context-dependence manner. Pharmacologic levels of butyrate act as an HDAC inhibitor that upregulates Annexin A1 and restrains ERK signaling, triggering apoptosis in PCa cells and exerting antiproliferative effects [219]. In contrast, under castration-resistant settings, low-millimolar acetate/butyrate can signal through TLR3 to induce PCa cell autophagy, activate NF-κB/MAPK, increase CCL20, and recruit/polarize macrophages toward an M2 phenotype, in a 362-patient cohort, higher intraprostatic CCL20 associated with adverse clinicopathologic features and shorter BCR-free survival [202]. Beyond local actions, SCFAs elevate systemic and intraprostatic IGF-1, thereby enhancing IGF1R/MAPK/PI3K signaling and fostering tumor growth in mouse models [203]. Among non-SCFAs metabolites, the microbial choline derivative TMAO promotes PCa cell proliferation/migration through p38 MAPK–driven HMOX1, and a high-choline diet augments tumor growth and pulmonary metastasis in vivo [174]. In contrast, phenylacetylglutamine suppresses PCa by upregulating CCNG2 and attenuating Wnt/β-catenin signaling [220]. Consistent with the gut–barrier–tumor axis, the *Akkermansia* metabolite inosine preserves epithelial integrity, lowers circulating LPS, and down-modulates tumor NF-κB/AR signaling to delay castration resistance [221]. Finally, the ellagitannin-derived metabolite urolithin A enhances NK cell cytotoxicity in PBMCs from men with PCa via AhR-linked mechanisms, supporting metabolite-based adjuvants to immunotherapy [204]. 

Clinically, gut dysbiosis elevates intratumoral LPS to activate the NF-κB/IL-6-STAT3 axis, driving proliferation and docetaxel resistance [222, 223]. In parallel, TLR9 expression in PCa associates with poor prognosis, reinforcing the value of co-profiling metabolite exposures with TLR/MAMP signatures in barrier-impaired states [224]. Collectively, these data argue for biomarker-guided, context-specific modulation of microbial pathways (e.g., SCFAs abundance, choline intake, barrier-stabilizing metabolites) to complement standard PCa therapies.

## Microbial metabolites at the helm: toward precision immune interventions

**Fig. 2:**
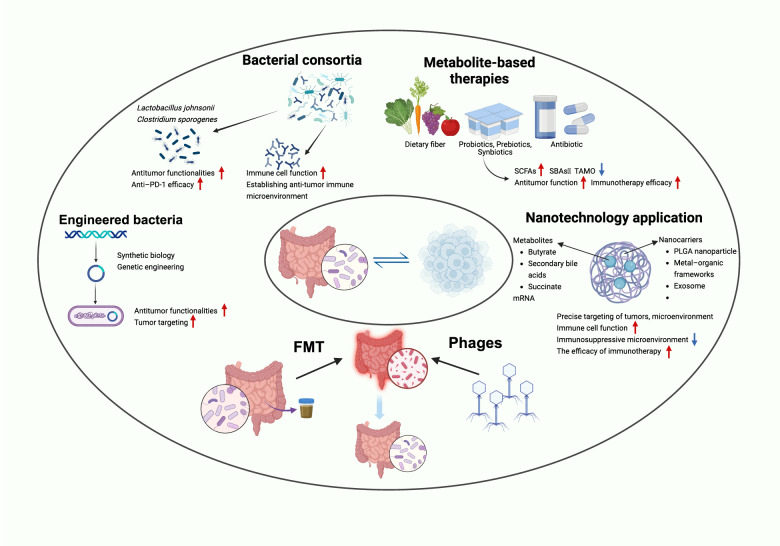
Strategies for Precision Immunotherapy via Modulation of Gut Microbiota-Derived Metabolites. This schematic illustrates the current landscape of precision strategies designed to reprogram antitumor immunity by targeting gut microbial metabolites. Microbiome reconstruction via fecal microbiota transplantation (FMT) or bacteriophage-based selective depletion; Engineered bacteria, employing synthetic biology to deliver tumor-targeted or immunoregulatory metabolites; Rational bacterial consortia, such as Lactobacillus johnsonii and Clostridium sporogenes, shown to improve CD8⁺ T-cell activity and bolster anti-PD-1 efficacy; Metabolite-based therapies using dietary fiber, prebiotics, or postbiotics to enrich beneficial metabolites while reducing immunosuppressive ones; Nanotechnology-based delivery systems, including PLGA nanoparticles, exosomes, and mRNA, enabling targeted metabolite delivery to the TME. Collectively, these modalities aim to restore immune homeostasis, reinvigorate cytotoxic responses, suppress immunosuppressive cell populations, and sensitize tumors to immune checkpoint blockade

Framed within the multifaceted axis of “metabolite-immune-tumor,” gut microbes exert profound influence on immune cell dynamics through their metabolic derivatives, intertwining themselves with tumorigenesis, disease evolution, and therapeutic reactivity. A paradigm shift is underway: from merely cataloging microbial signatures to synthetically engineering functional consortia and metabolite blueprints with the intent of recalibrating immune equilibrium and sustaining long-lasting antitumor responses. As visualized in Fig. 2, these next-generation precision immunotherapies hinge on modulating microbial metabolite flux, amplifying immunostimulatory mediators while silencing oncogenic metabolic circuits, to boost the potency and precision of anticancer responses.

### Microbiome-targeted modulation technologies

#### Fecal microbiota transplantation

FMT, which transfers gut microbiota from healthy donors to patients, has become a standard treatment for recurrent *Clostridioides* difficile infection [225]. After nearly seven decades of clinical practice and investigation, FMT has been recognized as a potential therapeutic modality for various disorders, including cancer [226]. FMT can restore gut microbial diversity and increase probiotic abundance in cancer patients, maintain microbiome homeostasis, enhance production of beneficial metabolites such as SCFAs, promote DCs maturation and antigen presentation, and bolster effector and memory differentiation of CD8⁺ T cells within the TME, thereby markedly improving antitumor immunity [24, 227–230]. Beyond modulating gut microbiota composition, FMT can reshape the TME to enhance immune cell infiltration and effector functions at the tumor site, thereby improving the overall efficacy of cancer immunotherapies. Clinical studies have demonstrated that in immunotherapy resistant melanoma patients, combining FMT with immune checkpoint inhibition therapy significantly increases CD8⁺ T cells, CD4⁺ T cells, and activated DCs within the TME, and upregulates Th1 cytokines such as IFN-γ and IL-12, thereby improving the body’s response rate to PD-1/CTLA-4 inhibitors [231, 232]. In addition, FMT selectively enriches beneficial strains such as *Bacteroides fragilis* and *B. thetaiotaomicron*, enhances IL-12-dependent Th1 immunity, and activates the STING signaling pathway, further strengthening antitumor immunity at the tumor site and reversing checkpoint inhibitor resistance [15]. With advances in novel FMT formulations, precision microbial component transplantation, and region-specific microbiota transfer, the prospects for tumor therapy are expanding; however, their clinical efficacy still requires further validation [226, 233]. 

#### Phage therapy

Beyond its traditional use against bacterial infections, phage therapy also holds great potential for remodeling the gut microbiota and its metabolome while eliciting systemic antitumor immunity. Phages selectively lyse overgrown or proinflammatory bacterial strains to reconstruct gut microbiota diversity, and via cascading effects regulate key metabolites such as SCFAs, SBAs, and aromatic amino acid derivatives, thereby mediating systemic immune modulation [234]. Engineered via synthetic biology, phages can be endowed with genes encoding metabolic enzymes or signaling factors, enabling precise modulation of the metabolome within the gut lumen [235]. Concurrently, phages can directly exert therapeutic effects by reshaping the TME and activating antitumor immune responses. Studies have shown that phages can activate DCs and enhance antigen presentation while suppressing MDSCs expansion within the tumor, thereby improving CD8⁺ T cell effector function and memory differentiation and remodeling the CRC immune microenvironment [236, 237]. In breast cancer models, engineered λ phages and M13 phage-based vaccines targeting the HER2 oncogene achieve tumor-specific recognition and elimination [238, 239]. Although phage therapy shows broad promise in cancer treatment, its safety and efficacy require validation in larger-scale clinical trials [240]. 

### Bacterial consortia and engineered bacteria

#### Bacterial consortia

Bacterial consortia are synergistic communities assembled by rigorous selection and rational pairing of multiple functionally complementary bacterial strains. Compared with FMT, bacterial consortia enable finer and more targeted modulation of the gut microbiota, thereby reducing adverse effects that may arise from whole-community transplantation [241]. The strains metabolically cooperate within the same niche and, via complementary metabolic pathways and immune signaling networks, collectively enhance the host’s antitumor immune response. Co-colonization with *Lactobacillus johnsonii* and *Clostridium sporogenes* synergistically produces IPA, promotes a T cell stem-like phenotype, and significantly enhances anti-PD-1 efficacy in melanoma, breast, and CRC models [107]. Symbiotic bifidobacterial consortia can modulate gut microbiota and immune cell distribution, enhance DCs antigen-presentation capacity, and promote activation and accumulation of CD8⁺ T cells within the TME, thereby improving antitumor immunity [242]. In one study employing an 11-strain defined consortia in germ-free mice, and found that the circulating metabolites produced by these 11 strains markedly increased infiltration and functionality of IFN-γ⁺CD8⁺ T cells in CRC and melanoma models, thereby establishing a favorable antitumor immune microenvironment [227]. Animal studies have demonstrated that a consortium of four Clostridium strains can both prevent tumor initiation and inhibit the growth of established tumors [243]. These studies indicate that bacterial consortia can be administered alone or in combination with chemotherapy, ICI, and other therapies to enhance host antitumor effects.

#### Engineered bacteria

Engineered Bacteria are microorganisms in which synthetic biology and genetic engineering have been applied to precisely edit specific genes and signaling circuits in native or model strains to confer or enhance antitumor functionalities [244]. One key mechanism is tumor targeting: by engineering genetic circuits in bacteria that respond to TME signals such as hypoxia, high lactate, or low pH (e.g., hypoxia-responsive promoters), or by expressing exogenous tumor-homing peptides (e.g., RGD), the strains selectively accumulate in tumors while minimizing invasion of healthy tissues [245]. Another mechanism is precise therapeutic payload delivery: bacteria utilize their type III secretion system or oncolytic proteins (e.g., listeriolysin O) to directly transfer antitumor proteins, nucleic acids, or small-molecule drugs into tumor cells, inducing anticancer effects via calcium overload or ferroptosis [246–248]. The third mechanism is remodeling the tumor immune microenvironment: engineered bacteria secrete a repertoire of signaling molecules, such as cytokines, chemokines, and metabolites, to finely tune the spatiotemporal dynamics of innate and adaptive immunity, markedly expand and activate effector subsets including CD8⁺ T cells and NK cells, and even biosynthesize checkpoint inhibitors in situ, thereby synergistically inducing solid tumor regression, inhibiting metastasis, and establishing durable immune memory [107, 248–250]. 

### Metabolite-based therapies and nanotechnology application

#### Metabolite-based therapies

Metabolite-based therapies encompass dietary interventions, supplementation with probiotics and prebiotics/synbiotics, and selective antibiotic modulation. For example, a high-fiber diet markedly increases SCFAs production and enhances cytotoxic T cell infiltration in the gut and tumors, thereby improving the sensitivity of melanoma-bearing mice to ICI therapy [21]. Moreover, this approach can inhibit the synthesis of SBAs, thereby exerting preventive and protective effects against CRC [251]. Simultaneously, it can also inhibit the metabolism of choline in intestinal microbiota, reduce the production of TMAO, and exert potential anti-tumor effects [252]. Supplementation with prebiotics such as inulin and pectin can decrease lactate levels and increase butyrate concentrations; these alterations modulate oncogenic and drug-resistance gene expression, thereby interfering with the growth of cancer cells and facilitating cancer treatment [253, 254]. 

Multiple classes of gut-derived metabolites can indeed be directly to achieve measurable and pharmacologically meaningful exposures systemically or in defined site (intestinal lumen/portal vein/TME), using routes that include intravenous infusion, oral pro-drugs or colon-targeted formulations, rectal perfusion/colonic release, and local (intratumoral/peritumoral) delivery. Proof-of-concept examples span several metabolite families. For one-carbon donors, intravenous sodium formate or oral formate pro-drug regimens raise circulating formate and are sufficient to augment de novo purine synthesis in tumor-infiltrating CD8⁺ T cells, thereby synergizing with anti-PD-1 and improving tumor control and survival in multiple murine models [52, 70, 72]. For purine nucleosides, oral supplementation of inosine enhanced the anti-tumor ability of T cells in mouse CRC, bladder cancer and melanoma models under the condition of combined IFN - γ co stimulation, and played a synergistic role with immunotherapy; peritumoral (intraperitoneal) dosing that increases local inosine also elevates intratumoral IFN-γ and constrains tumor growth [24, 180, 255]. For tryptophan catabolites, daily gavage with IPA (60 mg/kg) increases plasma and intratumoral IPA levels in multiple mouse transplant tumor models, boosts CD8⁺ T-cell infiltration/effector cytokines, and further enhances αPD-1 efficacy; similar anti-tumor effects can also be achieved by direct intratumoral injection of 5 µM IPA [107, 194]. Choline-derived TMAO shows similar “direct supplementation” feasibility: in triple-negative breast cancer, either oral TMAO/precursors or intratumoral injection TMAO reshapes the immune microenvironment, strengthens CD8⁺ effector function, and improves checkpoint inhibitor responses [178]. Classic SCFAs also fit this paradigm. Butyrate given orally or by systemic routes restrains tumor growth, improves cytotoxic T-cell programs, and can potentiate anti-PD-1 in several settings [14, 90–92, 256]. In humans, targeted SCFA delivery is feasible: acute intravenous or rectal SCFA administration produces quantifiable rises in circulating SCFAs accompanied by endocrine and immune changes (e.g., PYY/GLP-1 modulation and inflammatory cytokine changes) [257, 258]. Oral inulin-propionate ester achieves colonic propionate release with systemic metabolic and inflammatory effects in randomized trials—collectively supporting the translational plausibility of exposure-controlled “direct supplementation” strategies [259]. 

These data justify a route- and tissue-aware framework for metabolite therapy design: (i) systemic dosing (IV/oral pro-drug) when T-cell–intrinsic metabolic constraints are the target (e.g., formate-purine synthesis; inosine-ribose fueling); (ii) colon-targeted release or rectal perfusion when portal-vein or mucosal exposure is desired (e.g., SCFAs/endocrine–immune coupling); and (iii) intratumoral/peritumoral micro-dosing when high local concentrations with minimized systemic effects are preferable (e.g., IPA, TMAO).

#### Nanotechnology application

In recent years, researchers have encapsulated key microbial metabolites, such as SCFAs (e.g., butyrate), SBAs, succinate, and mRNA, into nanocarriers (PLGA nanoparticles, metal-organic frameworks, exosome, or lipid nanoparticle) to achieve precise targeting of the TME [260]. Loading succinate into tumor-derived exosomes drives TAMs toward an antitumor M1 phenotype via dual mechanisms of mitochondrial metabolic reprogramming and histone succinylation, significantly inhibiting tumor proliferation in liver, melanoma, lung, and breast cancer mouse models [261]. Furthermore, nanoparticle-mediated delivery of IDO1 inhibitors blocks the metabolic conversion of tryptophan to kynurenine, reverses the immunosuppressive microenvironment, and enhances intratumoral infiltration and effector activity of CD8⁺ T cells [262]. Another approach employs manganese-based nanomaterials to modulate fatty acid metabolism and induce ferroptosis in tumor cells, concurrently activating the cGAS-STING signaling pathway and enhancing the efficacy of immunotherapy [263]. These findings not only elucidate the molecular mechanisms by which nanocarriers reshape the tumor immune microenvironment through precise metabolite delivery but also lay a critical foundation for clinical translation.

### Clinical trials modulating the gut microbiome

A growing body of early-phase clinical evidence suggests that therapeutic remodeling of the gut microbiome can enhance immune checkpoint therapy. We searched the trial lists of ClinicalTrials.gov and NCI, and summarized the clinical studies combining microbiome interventions with ICI in Table 2 and Table S1. Two first-in-human studies in melanoma demonstrated that FMT from prior anti-PD-1 responders can induce microbiome engraftment, reprogram the TME, and convert a subset of anti-PD-1–refractory patients to clinical benefit—providing proof-of-concept that altering microbial ecosystems can modulate antitumor immunity in humans [230, 264]. A subsequent multicenter phase I trial brought FMT earlier into the treatment course, showing that healthy-donor, capsule-based FMT combined with first-line PD-1 blockade is feasible and biologically active [265]. Most recently, a multi-tumor study in patients refractory to PD-1 therapy reported sustained microbiome shifts and disease-control signals, further supporting the translatability of FMT beyond melanoma [266]. Together, these trials motivate systematic evaluation of who benefits, how to deliver FMT, and how to monitor on-treatment microbiome–immune dynamics.

**Table 2:** Ongoing clinical trials of gut Microbiome modulation combined with immune checkpoint inhibitors

Trial ID	Modality	Investigational product/donor	Tumor type & setting	ICI backbone/co-therapy	Phase & design	Current status (Oct 26, 2025)	Primary endpoint (key secondary)
NCT03353402	FMT (responder-derived)	Stool from ICI responders (single donor per cohort)	Metastatic melanoma, anti–PD-1–refractory	Pembrolizumab (reinduction)	Phase I, single-arm (proof-of-concept)	Completed/results published (Science 2021)	Safety/feasibility (ORR, immune & microbiome changes)
NCT03341143	FMT (responder-derived)	Stool from ICI responders (multiple donors)	Metastatic melanoma, anti–PD-1–refractory	Pembrolizumab (reinduction)	Phase I, single-arm	Completed/results published (Science 2021)	Safety/feasibility (ORR, PFS, immune profiling)
NCT03772899	FMT (healthy-donor)	Stool from rigorously screened healthy donors (oral caps.)	Advanced melanoma, first-line with anti–PD-1	Nivolumab or Pembrolizumab	Multicenter Phase I, single-arm	Completed/results published (Nat Med 2023)	Safety; (ORR, donor engraftment, correlative)
NCT04264975	FMT (responder-derived)	Stool from ICI responders	Multiple solid tumors, anti–PD-1–refractory	Anti–PD-1 (mostly pembrolizumab)	Phase I, single-arm	Completed/results published (Cell Host Microbe 2024)	Safety; signal of activity
NCT03829111	Live biotherapeutic product (LBP)	CBM588 (Clostridium butyricum) oral caps.	mRCC, first-line	Nivolumab + Ipilimumab	Randomized Phase I (2:1)	Completed/results published (Nat Med 2024)	Microbiome modulation (PFS, ORR)
NCT05122546	Live biotherapeutic product (LBP)	CBM588 (Clostridium butyricum)	mRCC, first-line	Cabozantinib + Nivolumab	Randomized Phase I (2:1)	Active/updated outcomes 2025 (GU symposium)	Alteration of Bifidobacterium spp. (PFS, ORR)
NCT05354102	Live biotherapeutic product (LBP)	BMC128 (4-strain rationally designed consortium)	Refractory NSCLC, RCC, melanoma	Nivolumab	Phase I, open-label (FIH)	Active, not recruiting; interim data (ASCO 2024)	Safety/tolerability (ORR; immune/microbiome)
NCT06540391	Live biotherapeutic product (LBP)	MB097 (precision microbiome co-therapy)	Advanced melanoma, primary resistance to anti-PD-1	Pembrolizumab	Phase Ib, randomized, open-label	Active, recruitment complete (MELODY-1)	Safety/engraftment (early efficacy)
NCT03637803	Live biotherapeutic product (LBP)	MRx0518 (Enterococcus faecium)	Multiple solid tumors, anti-PD-1–refractory	Pembrolizumab	Phase I/II	Terminated early (company administration)	Safety (ORR, biomarker)
NCT05821751	Dietary prebiotic	Inulin (prebiotic fiber)	R/M HNSCC on ICIs ± chemotherapy (PRINCESS)	Anti–PD-1/PD-L1 per SOC	Prospective, open-label (non-pharmacological)	Recruiting	Translational immune/microbiome endpoints (safety, ORR)
NCT06475807	Dietary (fiber & fermented foods/resistant starch)	High-fermented foods; high-fiber/resistant starch (sequential)	Stage IIB–IIIC melanoma; Stage IB–IIIC NSCLC receiving ICIs	Anti–PD-1/PD-L1 per SOC	Pilot, interventional (dietary)	Active	Microbiome changes (immune/metabolomic, clinical signals)
NCI-2024-01235	Dietary + ICI	Prebiotic-enriched diet	Unresectable refractory melanoma	Ipilimumab + Nivolumab	Phase II	Active (NCI listing)	Efficacy and safety
NCT04951583	FMT (healthy/responder, capsulized)	FMT capsules from responder donor	mRCC or NSCLC, first-line with ICIs	Pembrolizumab ± axitinib (per protocol)	Phase II, randomized	Ongoing	ORR; survival; microbiome engraftment
NCT05865730	Akkermansia-based LBP	Oncobax-AK (Akkermansia muciniphila, P2261)	ccRCC or NSCLC with low Akkermansia	Nivolumab + Ipilimumab (± others per cohort)	Phase I/IIa, multicenter	Ongoing; Phase 1 results presented (ASCO/AACR 2025)	Safety (efficacy signals; immune/metabolomic)
NCT06865521	Probiotic + ICI	Akkermansia probiotics (capsule)	Advanced solid tumors on anti-PD-1	Anti-PD-1 (unspecified)	Phase I, single-arm	Recruiting	Safety/feasibility
NCT04208958	LBP (defined consortium)	VE800 (Vedanta Biosciences)	Advanced/metastatic melanoma, MSS CRC, gastric/GEJ	Nivolumab (+ short course oral vancomycin pre-conditioning)	Phase 1/2, multicenter, open-label	Completed (per registry)	Safety; ORR

This table summarizes interventional human studies that combine gut microbiome–targeted strategies with immune checkpoint inhibitors (ICIs) to enhance antitumor immunity. We include four modality blocks—FMT (responder-derived or healthy-donor), live biotherapeutic products (LBPs; single-strain or defined consortia), and diet/prebiotic interventions—and list trials that are Recruiting, Active, Not yet recruiting, or Active (not recruiting) at the latest registry update. Consistent with this review’s narrative flow, trials first enumerate those discussed in the main text (in-text order), followed by additional ongoing studies sorted by start year.

Donor type (FMT): “Responder-derived” indicates donors with prior clinical benefit to anti-PD-1; “Healthy-donor” indicates rigorously screened non-cancer donors.“ICI backbone” records the PD-(L)1 and/or CTLA-4 regimen combined with the microbiome intervention. “Phase & design” uses brief descriptors (e.g., Phase I/II; randomized vs. single-arm). “Primary endpoint (key secondary)” reflects the registered primary outcome with a concise note on key secondaries relevant to immunotherapy (e.g., ORR, PFS, DOR, DCR, translational correlatives). Trial status and NCT numbers are taken from ClinicalTrials.gov or NCI listings.

*AE* Adverse event, *DCR* Disease control rate, *DOR* Duration of response, *FIH* First-in-human, *FMT* Fecal microbiota transplantation, *ICI* Immune checkpoint inhibitor, *LBP* Live biotherapeutic product, *NSCLC* Non-small-cell lung cancer, *ORR* Objective response rate, *PD-(L)1* Programmed cell death-(ligand) 1, *PFS* Progression-free survival, *RCC* Renal cell carcinoma, *SoC* Standard of care

Beyond FMT, several live biotherapeutic approaches are in progress. The spore-forming single strain *Clostridium butyricum* (CBM588) improved progression-free survival and yielded higher response rates when added to nivolumab/ipilimumab in a randomized phase I study of metastatic renal cell carcinoma, and showed a further clinical activity signal when combined with nivolumab plus cabozantinib as first-line therapy, supporting a plausible immunomodulatory contribution of microbiome tuning to ICI outcomes [267, 268]. Other defined consortia (e.g., BMC128, MB097) and single-strain products (e.g., MRx0518) are being evaluated with PD-1 backbones across melanoma, NSCLC and RCC, to link colonization, metabolite flux, and immune remodeling to patient outcomes [269–272]. Collectively, these studies extend the clinical armamentarium from donor-dependent FMT toward manufacturable, quality-controlled LBPs that may be easier to scale and regulate.

Dietary and prebiotic strategies affect gut microbiota and regulate cancer response to ICI therapy. Preclinical data suggest that a high-fiber diet correlates with improved anti-PD-1 outcomes and augments intratumoral immunity, prompting prospective trials that pair therapeutic dietary regimens or prebiotic supplements (such as inulin) with ICI in specific cancer treatments [21, 273, 274].

Across modalities, methodological heterogeneity remains a major determinant of outcome interpretation and cross-trial comparability. Key variables include donor/product selection, delivery route (capsule vs. endoscopic), antibiotic preconditioning, and the depth of on-treatment correlative profiling [275–277]. Of note, a randomized study testing vancomycin preconditioning before an oral Firmicutes formulation reported inferior responses in the antibiotic-conditioning arm, prompting caution regarding indiscriminate antibiotic use in this setting [278].

## Challenges and controversies 

Across solid tumors, gut microbial metabolites act via epigenetic remodeling, receptor signaling, metabolic rewiring, and an antigenic presentation arm to unconventional TCRs, these axes intersect differently across tissues and disease states, yielding divergent outcomes [20, 35, 36, 42, 43, 53]. 

Bidirectionality and tissue specificity. The same metabolite can promote or restrain antitumor immunity depending on dose, cell type, and organ context. SCFAs (C2-C4) can potentiate CD8⁺ T-cell metabolism and dendritic-cell competence, enhancing anti–PD-1 responses in defined settings [90–92], yet systemic SCFAs can blunt CTLA-4 blockade, curb NK-cell effector function, or favor myeloid immunosuppression via FFAR2⁺ MDSCs [53, 89, 103]. Formate (C1) not only fuel T-cell purine synthesis and enhance anti–PD-1 in vivo, but also promote tumor invasion and metastasis in some cases [72, 74]. Meanwhile, LPS lipid-A acylation states stratify ICI responsiveness: hexa-acylated LPS boosts anti–PD-1 efficacy whereas penta-acylated species do not and can even antagonize hexa-acylated signaling [156]. Separately, bacterial muropeptides that stimulate NOD2 can augment anti-PD-L1, while chemotherapy such as cyclophosphamide induces Gram-positive translocation to lymphoid tissues that amplifies Th17/CTL priming [37, 166]. These data argue that dosing route (luminal vs. systemic), tissue accumulation, and molecular speciation of the same “metabolite class” determine net immunologic directionality rather than metabolite identity alone. Moreover, these pattern-bound axes should be co-profiled with metabolite programs when interpreting mechanisms or selecting combinations, and unconventional T cell polarization should also be considered. 

Inter-individual heterogeneity. Microbiome composition, host genetics, diet, prior antibiotics, and disease stage variably shape metabolite fluxes and receptor wiring, complicating prediction of benefit vs. harm [18, 33, 42, 53, 202]. Clinically, the same pathway can correlate with response in one cohort but not another (e.g., tryptophan catabolites, TMAO), underscoring the need for biomarker-guided stratification rather than one-size-fits-all supplementation. 

Knowledge gaps in pharmacokinetic/biodistribution models and long-term safety data. For most metabolites and delivery formats, we still lack validated models that link input (dietary fiber, engineered consortia, nanoformulations) to in-tissue exposure and immune readouts, defining a therapeutic window is difficult [226, 234–240]. Moreover, ecological persistence and off-target risks of FMT, phages, and engineered strains remain incompletely characterized. Emerging biosensing and imaging tools promise continuous or spatially resolved metabolite tracking, but cancer-immunology use is nascent [279–282]. 

Uncertain clinical translational paths. Even for well-studied axes, translation can bifurcate: butyrate may potentiate PD-1 therapy yet limit CTLA-4 efficacy; [89–92] TMAO shows tumor-promoting programs in some contexts but improves ICB response in others [34, 174–178], and can be depleted by broad-spectrum antibiotics that also impair CAR-T potency [179]. These state-specific effects complicate trial design and clinical adoption. Safety and ecology remain moving targets for FMT, phage, and engineered strains; durable reprogramming without off-target immunosuppression is unresolved. 

## Future directions and conclusion 

Across solid tumors, gut-microbial metabolites act less as universal “good” or “bad” agents than as context-dependent rheostats that rewire innate and adaptive circuits. Identical metabolite classes can diverge by route of exposure, tissue accumulation, and molecular speciation. In parallel, pattern-bound MAMPs act as localized, structure-defined cues. Microbial metabolites activate unconventional T cells through TCR, and their polarity depends on ligand type, dosage, and tissue inflammation. These points argue for state matching that “how to give, where to give, and in what molecular form to give” often determines the direction of immunity more than “what metabolites to call”.

The next phase should move from correlative associations to causal, tissue-contextual maps that align metabolite flux with immune circuit rewiring and clinical endpoints across tumor types. Practically, this requires integrative multi-omics (shotgun metagenomics, targeted/untargeted metabolomics, lipidomics and immunomics) anchored to single-cell and spatial readouts, then learned jointly by machine-learning models to infer the “metabolite-immune-tumor connectome” and nominate druggable nodes for each patient [279, 281]. Emerging cross-platform pipelines that co-register imaging mass spectrometry (metabolites/lipids) with imaging mass cytometry (immune phenotypes) on the same tissue section provide a near-cellular view of metabolite heterogeneity and its alignment with immune neighborhoods, and should be adopted to benchmark solid-tumor atlases [283]. Beyond receptor signaling, for microbial metabolites of unconventional T cell antigens, we recommend analyzing the " ligandomics” layer through spatial metabolomics and pairing it with MR1/CD1d/BTN multiplex protein profiles and unconventional T cell status to guide combination therapy [30, 282]. Critically, metabolite programs should be profiled alongside “pattern-bound” MAMP axes that act locally and structure-specifically. In particular, lipid-A speciation analysis (such as the rapid lipid-A LC-MS/MS/MALDI workflow) can serve as a companion diagnosis in immunotherapy trials [284]. Likewise, NOD2-stimulatory muropeptides (e.g., from Enterococcus SagA remodeling) can enhance anti-PD-L1 efficacy in vivo, motivating muropeptide-sensor readouts to complement metabolite panels. 

To ensure translational relevance, prospective discovery–validation cohorts should pre-specify multi-omic endpoints, harmonized metadata, and use contamination-aware workflows from collection to sequencing/metabolomics, especially for low-biomass tumor samples, following the RIDE checklist and the Nature Microbiology 2025 consensus recommendations [41, 173]. Longitudinal biosensing to track systemic and intratumoral metabolite dynamics will be pivotal for dosing and for guarding against off-target immune effects. Recent advances include aptamer-based electrochemical biosensors for microbial targets/metabolites suitable for miniaturization and point-of-care deployment [280, 285], as well as spatiotemporal metabolomic imaging frameworks that can map metabolite gradients during therapy [286]. Operationally, this article proposes a translational framework: (i) non-invasive stool, plasma, tissue metabolite, and TCR-ligand panels for screening and dose titration; (ii) peri-operative or image-guided tissue sampling for spatial profiling at key timepoints, enabling closed-loop adjustment of fiber/probiotic/synbiotic regimens, metabolite mimetics, or nano-delivery schedules [263, 279, 280]. 

The key to clinical translation lies in defining the exposure effect window under different administration routes (intraluminal vs. systemic) and delivery forms (dietary fiber, defined microbiota, engineered bacteria, nanocarriers). We advocate combining stable-isotope tracing (including single-cell spatial isotope tracing) with population PK/PD modeling to calibrate tissue bioavailability and flux from dietary or synthetic inputs [287]. In parallel, trial logistics must incorporate contamination-aware workflows and negative controls from sample collection to sequencing and metabolomics to reduce false associations [41]. Building on this closed-loop framework, the focus of future research can be specifically summarized as four key questions that need to be prioritized for answer in the next 3–5 years: 

1. Clinical feasibility of biomarkers: can stool, plasma, tissue metabolite, and TCR-ligand panels achieve assay reproducibility, inter-lab calibration and clinical-grade cutoffs?
2. Personalized micro-ecological interventions: what algorithmic rules link baseline pathway activity to choice of fiber/synbiotic formula, engineered consortia, or nano-formulated mimetics?
3. Safety and ecological stability: what persistence and horizontal-gene-transfer risks arise with FMT, phages, and engineered strains, and how do we monitor ecological drift post-therapy?
4. Manufacturing and access—what QC and release criteria are needed for Good Manufacturing Practice of metabolite formulations and living biotherapeutics to ensure batch-to-batch functional equivalence?

Ultimately, it is the synergy of context-aware mechanism, precision delivery, stringent biosafety, and integrative analytics that will carry microbially derived metabolite circuits from concept to clinic.

## Supplementary Information

Supplementary Material 1

## Data Availability

No datasets were generated or analysed during the current study.

## Abbreviations

AhR: Aryl hydrocarbon receptor
AMPK: AMP-activated protein kinase
APC: Antigenpresenting cell
CAR-T: Chimeric antigen receptor T cell
CCA: Cholangiocarcinoma
CRC: Colorectal cancer
CTL: Cytotoxic T lymphocyte
CXCR: C-X-C chemokine receptor
DCs: Dendritic cells
DCA: Deoxycholic acid
FFAR2: Free fatty acid receptor 2
FMT: Fecal microbiota transplantation
GPCR: G protein-coupled receptors
HCC: Hepatocellular carcinoma
ICB: Immune checkpoint blockade
ICI: Immune checkpoint inhibitor
IDO: Indoleamine 23dioxygenase
ILA: Indole3lactic acid
iNKT: Invariant NKT
IPA: Indole3propionic acid
IPyA: Indole3pyruvic acid
LCA: Lithocholic acid
LPS: Lipopolysaccharide
MAMPs: Microbeassociated molecular patterns
MDSCs: Myeloidderived suppressor cells
NK: Natural killer cell
NKT: Natural killer T cell
NOD: Nucleotidebinding oligomerization domain
NSCLC: Nonsmall cell lung cancer
PCa: Prostate cancer
PDAC: Pancreatic ductal adenocarcinoma
PPAR: Peroxisome proliferatoractivated receptor
PRR: Pattern recognition receptor
ROS: Reactive oxygen species
SBAs: Secondary bile acids
SCFAs: Shortchain fatty acids
STAT3: Signal transducer and activator of transcription 3
TAMs: Tumorassociated macrophages
TCR: T-cell receptor
TLCA: Taurolithocholic acid
TLR4: Tolllike receptor 4
TMAO: Trimethylamine Noxide
TME: Tumor microenvironment
Tregs: Regulatory T cells
TNBC: Triplenegative breast cancer
UDCA: Ursodeoxycholic acid
3-IAA: Indole3acetic acid
